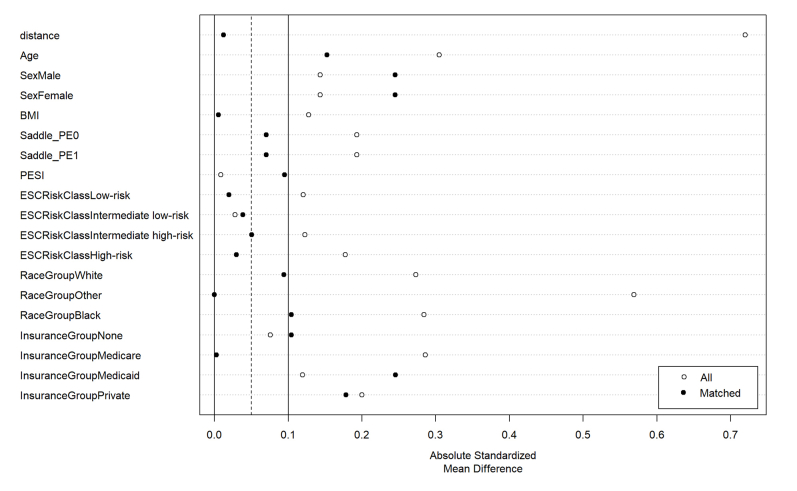# Outcomes of patients with acute pulmonary embolism managed in-house vs those transferred between hospitals: a retrospective observational study

**DOI:** 10.1016/j.rpth.2024.102606

**Published:** 2024-10-29

**Authors:** Priyanka Sridhar, Hong Yu Wang, Agostina Velo, Destiny Nguyen, Avinash Singh, Abdul Rehman, Jason Filopei, Madeline Ehrlich, Robert Lookstein, David J. Steiger

**Affiliations:** 1 Department of Medicine, Icahn School of Medicine at Mount Sinai Health System, New York City, New York, USA; 2 Division of Pulmonary, Critical Care, and Sleep Medicine, Department of Medicine, Icahn School of Medicine at Mount Sinai Health System, New York City, New York, USA; 3 Department of Medicine, TidalHealth Peninsula Regional, Salisbury, Maryland, USA; 4 Department of Radiology, Icahn School of Medicine at Mount Sinai Health System, New York City, New York, USA

**Keywords:** bleeding, embolectomy, length of stay, mortality, patient transfer, pulmonary embolism

## Abstract

**Background:**

Interhospital transfer (IHT) for acute pulmonary embolism (PE) is increasingly performed to improve access to advanced reperfusion therapies. It is unclear if outcomes of patients undergoing IHT are comparable with those of patients presenting in-house to hospitals with PE Response Team (PERT) capabilities.

**Objectives:**

To determine whether outcomes of patients with acute PE undergoing IHT differ from those of patients presenting in-house.

**Methods:**

We retrospectively reviewed 386 patients with acute PE who were treated by PERT at 1 of 3 urban teaching hospitals in the Mount Sinai Health System in New York City from January 2021 to October 2023. Propensity score–weighted analysis was performed to compare the outcomes of patients managed in-house with those of patients undergoing IHT.

**Results:**

Two hundred eighty-four patients presented in-house, while 102 were transferred from other hospitals. Median PE Severity Index score was 84, and 3 (0.8%), 80 (20.7%), 237 (61.4%), and 66 (17.1%) had low-risk, intermediate low–risk, intermediate high–risk, and high-risk PE. Odds of receiving systemic thrombolysis (odds ratio [OR], 1.06; *P* = .06) or advanced therapies (OR, 0.95; *P* = .003) were not significantly different between the 2 groups. Rates of 30-day mortality, major bleeding, and readmission were 6.9%, 2.9%, and 9.8% for the IHT group and 10.6%, 2.1%, and 13% for the in-house group, respectively. IHT patients had lower odds of 30-day mortality (OR, 0.88; *P* = .003) and higher odds of major bleeding (OR, 1.03; *P* = .04).

**Conclusion:**

PERT-guided IHT for patients with acute PE was associated with reduced mortality but increased risk of bleeding compared with patients managed in-house at hospitals with PERT capabilities.

## Essentials

• Outcomes of interhospital transfer (IHT) for pulmonary embolism remain unexplored.
• Retrospective review of patients with acute pulmonary embolism (102 IHT vs 284 managed in-house) was performed.
• IHT patients had lower odds of 30-day mortality and higher odds of major bleeding.
• Odds of receiving advanced therapies were not significantly different between the 2 groups.

## Graphical abstract

**Figure:**
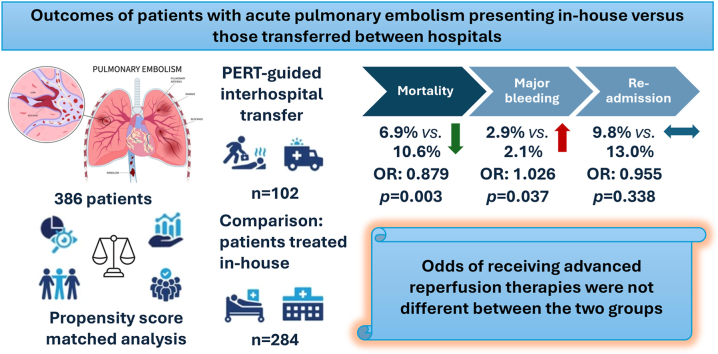

## 1 Introduction

Pulmonary embolism (PE) is one of the leading causes of preventable cardiovascular mortality [1], and up to 50% of acute PE survivors experience long-term sequelae—attributable to the post-PE syndrome—despite adequate anticoagulation [2]. Chronic, slowly resolving thrombi cause persistent pulmonary vascular and right ventricular (RV) dysfunction, which can lead to chronic thromboembolic pulmonary hypertension as well as have profound impacts on patients’ quality of life. Advanced reperfusion therapies—such as catheter-directed thrombolysis (CDT) and catheter-directed embolectomy (CDE)—acutely reduce clot burden, lower pulmonary vascular resistance, improve RV function, and positively influence quality of life [3]. Catheter-directed and surgical reperfusion therapies are only available at hospitals with critical care and surgical capabilities and experienced healthcare providers who can manage patients undergoing advanced reperfusion therapies and/or requiring mechanical circulatory support [4]. PE is a complex clinical entity with both acute and chronic major morbidity and mortality, and therefore, appropriate management is facilitated by multidisciplinary care provided by a robust PE Response Team (PERT).

The European Society of Cardiology (ESC), in its 2019 guidelines on the diagnosis and management of acute PE, has emphasized the importance of PERT for the management of patients with intermediate- and high-risk PE [5]. Triaging patients and making lifesaving decisions regarding the need for advanced reperfusion therapies is reliant on the presence of facilities with access to such care. Patients arriving at hospitals that lack these expert facilities need to be transferred to centers of advanced care, and these time-sensitive decisions are usually guided by PERT at the receiving hospital [4]. Communication, team efforts, appropriate indications, and operational factors can serve as both facilitators and barriers to interhospital transfer (IHT), which can ultimately influence patient outcomes [6]. Only a few studies have compared the outcomes of acute PE patients treated in-house with those of patients transferred from other facilities [[7], [8], [9], [10]]. Among the published studies, the IHT group has been shown to have shorter PERT activation times, faster intervention times, and an overall higher likelihood of receiving advanced therapies compared with direct admits; however, the rates of mortality between the 2 groups have been variable [[7], [8], [9], [10], [11], [12], [13]]. In a previous study of patients undergoing IHT for acute PE, we observed that the time taken to achieve IHT was not associated with receipt of advanced therapies for PE or overall patient mortality [11].

IHT for acute PE is increasingly being performed to improve access to reperfusion therapies. Data from the PERT Consortium database showed that as of March 1, 2022, a quarter of studied patients had undergone IHT for advanced therapies [14]. However, there is a dearth of literature comparing the outcomes of patients who undergo IHT for acute PE with those of patients who are treated in-house [[7], [8], [9], [10]]. In the present study, we compared the outcomes of acute PE patients who underwent IHT with those of patients who presented directly to a hospital with PERT capabilities. By performing a comparative analysis, we hoped to bridge the evidence gap in the present literature regarding outcomes of IHT for acute PE.

## 2 Methods

After obtaining approval from the institutional review board, a retrospective cross-sectional study was performed. Given that we only utilized deidentified patient data and no physical patient contact was involved, the requirement of informed consent was waived for this retrospective observational study. We included patients who had been treated by PERT for acute PE from January 2021 to October 2023 at 1 of 3 urban teaching hospitals in the Mount Sinai Health System (New York, New York)—Mount Sinai Beth Israel, Mount Sinai West, and Mount Sinai Morningside. Patients who had missing data on primary outcomes were excluded from the study. We also excluded IHT patients who did not complete the transfer process or underwent IHT for indications other than acute PE. Of the patients who did not complete transfer (*n* = 9), none of the patients died; the reasons for noncompletion of transfer were patient or family refusal (*n* = 2) and lack of beds (*n* = 7). PERT-activated PE patients were divided into 2 groups: the “IHT” group (patients who had been transferred from outside hospitals) and the “in-house” or the “control” group (patients who presented directly to our hospitals).

### 2.1 PERT and IHT

In the Mount Sinai Health System, all patients with intermediate- to high-risk acute PE were managed by a multidisciplinary PERT. For patients undergoing IHT, all patients were discussed with the PERT team and accepted by the PERT team prior to transfer. In rare cases, PERT consultation could be requested for patients with “low-risk” acute PE if there were significant medical comorbidities, severe concurrent illness, and/or high clot burden with impending risk for further thromboembolism. A detailed description of the PERT has been published previously [15]. The PERT consisted of the on-call intensivist, interventional radiologist, cardiothoracic surgeon, cardiologist, and pulmonologist. A PERT consult for “in-house” patients could be initiated by the patient’s primary attending. For each PERT consult, a discussion was held between members of the PERT and the patient’s primary attending, and a mutual plan of care was developed. In cases of IHT, PERT consultation was requested by treating physicians at outside hospitals through the patient access center. The general criteria for requesting a PERT consultation for IHT was the diagnosis of intermediate- to high-risk acute PE at a hospital without availability of PERT expertise or access to advanced therapies for acute PE. In these cases, the final decision as to whether an IHT was medically indicated was made by mutual discussion between members of the PERT and the patient’s treating physician. Most PERT consultations for IHT were requested from smaller affiliated hospitals within the Mount Sinai Health System, although out-of-system transfers from other hospitals were also included. All hospitals from which patients were transferred were located within a 75-mile radius. Ground transportation was mostly utilized for transferring patients from the referring institution to the receiving hospital, although in rare cases, air ambulances were also used.

In all patients, a diagnosis of acute PE was confirmed by visualizing filling defects within the pulmonary arteries on contrast-enhanced computed tomography scans. Risk stratification of patients with acute PE was based on guidelines from the ESC [5]. In brief, patients with hemodynamically significant PE were classified as high-risk. Patients with normal hemodynamics but evidence of RV dysfunction and elevated biomarkers (ie, brain natriuretic peptide or troponin) were deemed intermediate high–risk. Patients with either elevated biomarkers or evidence of RV dysfunction—but not both—with normal hemodynamics were classified as intermediate low–risk. Patients who had stable hemodynamics, normal biomarkers, and absence of RV dysfunction were deemed low-risk. In the absence of contraindications, therapeutic anticoagulation was administered to all patients diagnosed with acute PE.

### 2.2 Advanced reperfusion therapies

Advanced reperfusion therapies offered to patients within the Mount Sinai Health System included CDT, CDE, and surgical embolectomy (SE). CDT was performed by percutaneously establishing central venous access, placing catheters within bilateral main pulmonary arteries, and infusing alteplase slowly over 24 hours. CDE was performed percutaneously using FlowTriever (Inari Medical), a mechanical suction thrombectomy device. SE was performed using a midline sternotomy approach followed by total cardiopulmonary bypass and direct excision of clots from the pulmonary trunk. General indications for SE included radiographic signs of chronic thromboembolic pulmonary hypertension, failed CDE or CDT, and significant hemodynamic instability precluding catheter interventions.

### 2.3 Data collection

Eligible patients were identified by retrieving hospital records for all patients with an International Classification of Diseases, Tenth Revision, Clinical Modification diagnosis of acute PE and the list of PERT consultations for IHT during the study period. Medical records for all eligible patients were systematically reviewed, and data pertaining to demographics, clinical features, medical history, treatment, primary outcomes, and outpatient follow-up were recorded. Data were initially recorded in Microsoft Excel spreadsheets and later imported into statistical software for analysis. Primary outcomes included in-hospital mortality, in-hospital bleeding, 30-day mortality, 30-day bleeding, and 30-day readmission. We also extracted information pertaining to the receipt of advanced reperfusion therapies. Racial group membership was based on patient self-report, while insurance information was extracted from the electronic health record and billing records.

### 2.4 Statistical analysis

Statistical analysis was performed in R version 4.1.1 (R Project for Statistical Computing). For qualitative variables, frequencies were computed, while median and IQR were computed for quantitative variables. Univariable comparison of quantitative variables between the IHT and the in-house groups was performed using independent samples *t*-test, while univariable comparison of qualitative variables between the 2 groups was performed using either chi-square or Fisher’s exact test. A propensity score–matched analysis was performed using the *MatchIt* package for R. A propensity score was calculated using a generalized linear model with the *probit* link function from the following 8 variables: age, biological sex (assigned at birth), body mass index (BMI), race, insurance, PE Severity Index (PESI) score, ESC risk class, and presence of a saddle embolus. Matching on the propensity score was performed using the optimal *full* matching specification with the target estimand being “ATT” (average treatment effect in the treated). An adequate balance between the 2 groups (IHT vs in-house) was ensured by examining density plots and standardized mean differences (see Supplementary Table S1 and Supplementary Figure S1). Quasi-binomial regression models were used to compare the outcomes of mortality, bleeding, readmission, and follow-up between the 2 groups. A negative binomial regression model was used to compare the hospital length of stay (LOS) between the 2 groups. Cluster robust SEs were calculated using the *marginal effects* package for R. For multivariable regression models constructed in the propensity score–matched sample, all covariates (matching variables) were included as interaction terms for a “doubly robust” estimate. All *P* values were adjusted for multiple comparisons using the modified Bonferroni procedure described by Hochberg [16]. A *P* value of less than .05 was considered statistically significant for all comparisons.

## 3 Results

**Figure 1:**
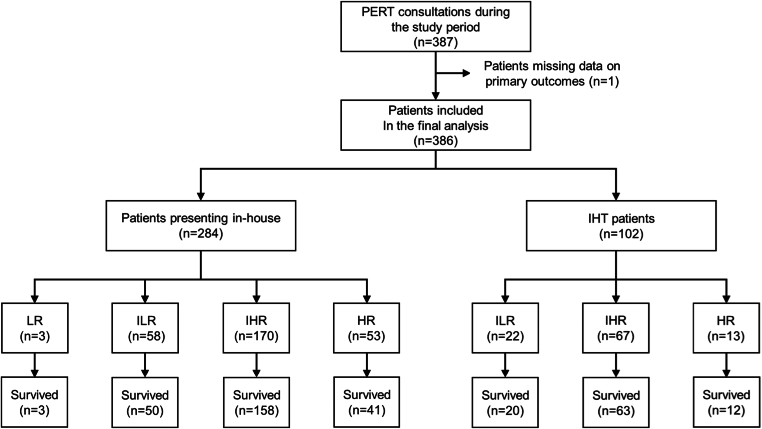
A flowchart depicting the overall outcome of patients presenting in-house vs those transferred from outside hospitals. HR, risk; IHR, intermediate high risk; IHT, interhospital transfer; ILR, intermediate low risk; LR, low risk; PERT, pulmonary embolism response team. Survived denotes 30 days following discharge from the hospital.

During the study period, 387 patients were eligible for inclusion, of which 1 patient was excluded due to missing data on primary outcomes. This left a total of 386 patients to be included in the final analysis; of these, 102 (26.4%) were transferred from other hospitals (the IHT group), and 284 (73.6%) had presented in-house (see Figure 1).

The median age of included patients was 64 (IQR, 52-75) years, and there was a slight preponderance of female patients (*n* = 207; 53.6%). Median BMI of included patients was 28.4 (IQR, 24.4-34.9) kg/m^2^. With respect to racial group membership, White (*n* = 201; 52.1%) and Black (*n* = 142; 36.8%) patients constituted the bulk of the sample (see Table 1). Most patients had some form of medical insurance, with the most common being private insurance (*n* = 120; 31.1%), followed by government Medicare (*n* = 94; 24.3%) and commercial Medicare (*n* = 77; 19.9%). Only 15 (3.9%) patients did not have any medical insurance.

**Table 1:** Demographic, clinical, laboratory, and radiologic features of patients included in the study (*N* = 386).

Characteristics	Overall (*N* = 386)	In-house (*n* = 284)	IHT (*n* = 102)
Age (y), median (IQR)	64 (52-75)	65 (53-76)	44 (60-72)
Sex			
Female	207 (53.6%)	147 (51.8%)	60 (58.8%)
Male	179 (46.4%)	137 (48.2%)	42 (41.2%)
Race			
White	212 (54.9%)	158 (55.6%)	54 (52.9%)
Black	149 (38.6%)	114 (40.1%)	35 (34.3%)
Asian	15 (3.9%)	10 (3.5%)	5 (4.9%)
American Indian	1 (0.3%)	1 (0.3%)	0 (0.0%)
Other	3 (0.8%)	1 (0.3%)	2 (2.0%)
Middle-Eastern	1 (0.3%)	0 (0.0%)	1 (1.0%)
Multiracial	5 (1.3%)	0 (0.0%)	5 (4.9%)
Insurance			
Government Medicare	94 (24.3%)	75 (26.4%)	19 (18.6%)
Government Medicaid	35 (9.1%)	21 (7.4%)	14 (13.7%)
Commercial Medicare	77 (19.9%)	61 (21.5%)	16 (15.7%)
Commercial Medicaid	45 (11.7%)	34 (12.0%)	11 (10.8%)
Private insurance	120 (31.1%)	81 (28.5%)	39 (38.2%)
None	15 (3.9%)	12 (4.2%)	3 (2.9%)
Body mass index (kg/m^2^), median (IQR)	28.4 (24.4-34.9)	28.1 (24.3-34.4)	29.4 (24.8-36.0)
CCI, median (IQR)	4 (1-6)	4 (2-6)	3 (1-6)
Past medical history			
Prior episode of VTE	78 (20.2%)	58 (20.4%)	20 (19.6%)
On anticoagulation	19 (4.9%)	11 (3.9%)	8 (7.8%)
Cardiac disease	35 (9.1%)	15 (5.3%)	20 (19.6%)
Asthma or COPD	56 (14.5%)	45 (15.8%)	11 (10.8%)
Other chronic lung disease	4 (1.0%)	0 (0.0%)	4 (3.9%)
Pulmonary hypertension	12 (3.1%)	6 (2.1%)	6 (5.9%)
Type of prior anticoagulant			
DOAC	9 (2.3%)	6 (2.1%)	3 (2.9%)
Warfarin	3 (0.8%)	2 (0.7%)	1 (1.0%)
LMWH	6 (1.5%)	3 (1.1%)	3 (2.9%)
Missing data	1 (0.3%)	0 (0.0%)	1 (1.0%)
Risk factors for PE			
Active malignancy	58 (15.0%)	50 (17.6%)	8 (7.8%)
Immobility for ≥3 d	31 (8.0%)	23 (8.1%)	8 (7.8%)
Surgery within 4 wk	33 (8.5%)	19 (6.7%)	14 (13.7%)
Active smoker	48 (12.4%)	31 (10.9%)	17 (16.7%)
Predisposing medications	22 (5.7%)	11 (3.9%)	11 (10.8%)
Recent long flight or travel	28 (7.2%)	17 (6.0%)	11 (10.8%)
Symptoms			
Pleuritic chest pain	152 (39.4%)	103 (36.3%)	49 (48.0%)
Hemoptysis	15 (3.9%)	10 (3.5%)	5 (4.9%)
Dyspnea	292 (75.6%)	203 (71.5%)	89 (87.2%)
Syncope	86 (22.3%)	59 (20.8%)	27 (26.5%)
Unilateral leg swelling	80 (20.7%)	59 (20.8%)	21 (20.6%)
Clinical signs			
Tachycardia	219 (56.7%)	152 (53.5%)	67 (65.7%)
Low systolic blood pressure	12 (3.10%)	5 (1.8%)	7 (6.9%)
Tachypnea	180 (46.6%)	138 (48.6%)	42 (41.2%)
Hypoxia	55 (14.2%)	19 (6.7%)	36 (35.3%)
PESI score, median (IQR)	84 (63-105)	82 (63-103)	86 (63-109)
ESC risk group			
Low risk	3 (0.8%)	3 (1.1%)	0 (0.0%)
Intermediate-low risk	80 (20.7%)	58 (20.4%)	22 (21.6%)
Intermediate-high risk	237 (61.4%)	170 (59.9%)	67 (65.7%)
High risk	66 (17.1%)	53 (18.7%)	13 (12.7%)
Site where PE diagnosed			
Emergency department	351 (90.9%)	260 (91.6%)	91 (89.2%)
Inpatient	35 (9.1%)	24 (8.4%)	11 (10.8%)
Disposition on admission			
Intensive care unit	297 (76.9%)	256 (90.1%)	41 (40.2%)
Step down unit	28 (7.2%)	0 (0.0%)	28 (27.4%)
Telemetry ward	12 (3.1%)	1 (0.3%)	11 (10.8%)
Medical or surgical floor	49 (12.7%)	27 (9.5%)	22 (21.6%)
Location of PE			
Saddle (central)	85 (22.0%)	56 (19.7%)	29 (28.4%)
Main pulmonary artery (central)	196 (50.8%)	126 (44.4%)	70 (68.6%)
Lobar branch (central)	265 (68.6%)	186 (65.5%)	79 (77.4%))
Segmental branch (peripheral)	213 (55.2%)	136 (47.9%)	77 (75.5%)
Subsegmental (peripheral)	147 (38.1%)	101 (35.6%)	46 (45.1%)
RV dilatation on CT	333 (86.3%)	247 (87.0%)	86 (84.3%)
Echocardiography performed	370 (95.8%)	270 (95.1%)	100 (98.0%)
RV dysfunction on TTE	288 (74.6%)	212 (74.6%)	76 (74.5%)
Venous Doppler study performed	300 (77.7%)	220 (77.5%)	80 (78.4%)
Findings of venous Doppler			
CFV thrombosis	58 (15.0%)	36 (12.7%)	22 (21.6%)
SFV thrombosis	21 (5.4%)	19 (6.7%)	2 (2.0%)
Popliteal vein thrombosis	80 (20.7%)	56 (19.7%)	24 (23.5%)
Other distal DVT	11 (2.8%)	9 (3.2%)	2 (2.0%)
Superficial vein thrombosis	3 (0.8%)	2 (0.7%)	1 (1.0%)
Laboratory investigations, median (IQR)			
D-dimer (mg/L)	11.0 (5.2-20.0)	10.5 (5.0-20.0)	19.9 (6.2-20.0)
Troponin I (ng/mL)	0.20 (0.06-1.10)	0.23 (0.06-2.26)	0.16 (0.05-0.44)
Brain natriuretic peptide (pg/mL)	170 (54-417)	155 (49-342)	225 (88-466)

Data are presented as *n* (%), unless stated otherwise.

CCI, Charlson Comorbidity Index; CFV, common femoral vein; COPD, chronic obstructive pulmonary disease; CT, computed tomography; DOAC, direct oral anticoagulant; DVT, deep venous thrombosis; ESC, European Society of Cardiology; IHT, interhospital transfer; LMWH, low-molecular-weight heparin; PE, pulmonary embolism; PESI, Pulmonary Embolism Severity Index; RV, right ventricle; SFV, superficial femoral vein; TTE, transthoracic echocardiography; VTE, venous thromboembolism.

The details of clinical features for our patient cohort are provided in Table 1. In brief, 219 (56.7%), 180 (46.6%), and 55 (14.2%) patients had tachycardia, tachypnea, and hypoxia on presentation. The median PESI score was 84 (IQR, 63-105) points. Based on ESC risk stratification, 3 (0.8%), 80 (20.7%), 237 (61.4%), and 66 (17.1%) patients had low-risk, intermediate low–risk, intermediate high–risk, and high-risk PE, respectively. Saddle, main pulmonary artery, lobar, segmental, and subsegmental PE were found in 85 (22.0%), 196 (50.8%), 265 (68.6%), 213 (55.2%), and 147 (38.1%) cases, respectively. Evidence of RV dilatation was evident on 333 (86.3%) computed tomography scans, while RV dysfunction was noted by echocardiography in 288 (74.6%) patients. Median levels of D-dimer, brain natriuretic peptide, and troponin I for our patients were 11.0 (IQR, 5.2-20.0) mg/L, 170 (IQR, 54-417) pg/mL, and 0.20 (IQR, 0.06-1.10) ng/mL, respectively. Further details can be found in Table 1.

With respect to treatment, systemic anticoagulation was administered to nearly all patients (*n* = 376; 97.4%). Systemic thrombolysis was administered to 22 (5.7%) patients, while inferior vena cava filter insertion was performed in 46 (11.9%) cases. With respect to advanced reperfusion therapies, 17 (4.4%), 43 (11.1%), and 8 (2.1%) patients received CDT, CDE, and SE, respectively. In terms of overall outcomes, the rates of in-hospital mortality and 30-day mortality were 6.5% (*n* = 25) and 9.6% (*n* = 37), respectively. In-hospital bleeding of any severity occurred in 32 (8.3%) cases, while 30-day bleeding of any severity occurred in 36 (9.3%) cases. In-hospital major bleeding and major bleeding at 30 days only occurred in 8 (2.1%) and 9 (2.3%) patients, respectively. The median hospital LOS was 6.2 (IQR, 3.6-10.8) days, and the rate of 30-day readmission was 12.2%. After discharge, rates of follow-up—at a 3-month interval—in primary care, pulmonary, and hematology clinics were 71.2% (*n* = 275), 30.3% (*n* = 117), and 20.5% (*n* = 79), respectively. Further details are provided in Table 2.

**Table 2:** Details of treatment and overall clinical outcomes of patients included in the study (*N* = 386).

Characteristics	Overall (*N* = 386)	In-house (*n* = 284)	IHT (*n* = 102)
Systemic anticoagulation	376 (97.4%)	280 (98.6%)	96 (94.1%)
Initial anticoagulant			
Unfractionated heparin	302 (78.2%)	220 (77.5%)	82 (80.4%)
Low-molecular-weight heparin	71 (18.4%)	58 (20.4%)	13 (12.7%)
DOAC	3 (0.8%)	2 (0.7%)	1 (1.0%)
IVC filter insertion	46 (11.9%)	39 (13.7%)	7 (6.9%)
Systemic thrombolysis	22 (5.7%)	13 (4.6%)	9 (8.8%)
Catheter-directed thrombolysis	17 (4.4%)	15 (5.3%)	2 (2.0%)
Catheter-directed embolectomy	43 (11.1%)	28 (9.9%)	15 (14.7%)
Surgical embolectomy	8 (2.1%)	7 (2.5%)	1 (1.0%)
Any advanced therapy	67 (17.4%)	49 (17.2%)	18 (17.7%)
Anticoagulant prescribed upon discharge			
Apixaban	250 (64.8%)	165 (58.1%)	85 (83.3%)
Dabigatran	6 (1.5%)	6 (2.1%)	0 (0.0%)
Rivaroxaban	34 (8.8%)	29 (10.2%)	5 (4.9%)
Edoxaban	1 (0.3%)	0 (0.0%)	1 (1.0%)
Warfarin	15 (3.9%)	12 (4.2%)	3 (2.9%)
Low-molecular-weight heparin	26 (6.7%)	23 (8.1%)	3 (2.9%)
None	32 (8.3%)	30 (10.6%)	2 (2.0%)
Overall outcomes			
Length of stay, median (IQR), d	6.2 (3.6-10.8)	6.2 (3.7-10.5)	5.9 (3.2-10.9)
In-hospital bleeding of any severity	32 (8.3%)	22 (7.7%)	10 (9.8%)
In-hospital major bleeding	8 (2.1%)	5 (1.8%)	3 (2.9%)
In-hospital death	25 (6.5%)	20 (7.0%)	5 (4.9%)
Death by 30 d	37 (9.6%)	30 (10.6%)	7 (6.9%)
30-d bleeding of any severity	36 (9.3%)	25 (8.8%)	11 (10.8%)
30-d major bleeding	9 (2.3%)	6 (2.1%)	3 (2.9%)
30-d readmission	47 (12.2%)	37 (13.0%)	10 (9.8%)
Cause of in-hospital death			
PE-related	11 (2.8%)	8 (2.8%)	3 (2.9%)
Other	14 (3.6%)	12 (4.2%)	2 (2.0%)
Cause of readmission			
Thrombosis	9 (2.3%)	8 (2.8%)	1 (1.0%)
Bleeding	4 (1.0%)	3 (1.1%)	1 (1.0%)
Other	34 (8.8%)	26 (9.1%)	8 (7.8%)
Outpatient follow-up			
Primary care	275 (71.2%)	216 (76.1%)	59 (57.8%)
Pulmonary	117 (30.3%)	75 (26.4%)	42 (41.2%)
Hematology	79 (20.5%)	41 (14.4%)	38 (37.2%)

Data are presented as *n* (%).

DOAC, direct oral anticoagulant; IHT, interhospital transfer; IVC, inferior vena cava; PE, pulmonary embolism.

### 3.1 Comparison of outcomes among the IHT and in-house groups

The IHT group consisted of 102 (26.4%) patients with a median PESI score of 86 (IQR, 63-109) points, while the in-house group consisted of 284 (73.6%) patients with a median PESI score of 82 (63-103) points. With respect to ESC risk stratification, 22 of 102 (21.6%), 67 of 102 (65.7%), and 13 of 102 (12.7%) patients had intermediate low–risk, intermediate high–risk, and high-risk PE in the IHT group, respectively, while 3 of 284 (1.1%), 58 of 284 (20.4%), 170 of 284 (59.9%), and 53 of 284 (18.7%) patients had low-risk, intermediate low–risk, intermediate high–risk, and high-risk PE in the in-house group, respectively (see Figure 1). Based on univariable analyses, no major significant differences were noted between the IHT and the in-house groups in terms of demographics (except race), baseline characteristics (except disposition on admission), and clinical features (except hypoxia on presentation)—details of these statistical results are provided in Supplementary Table S2.

**Figure 2:**
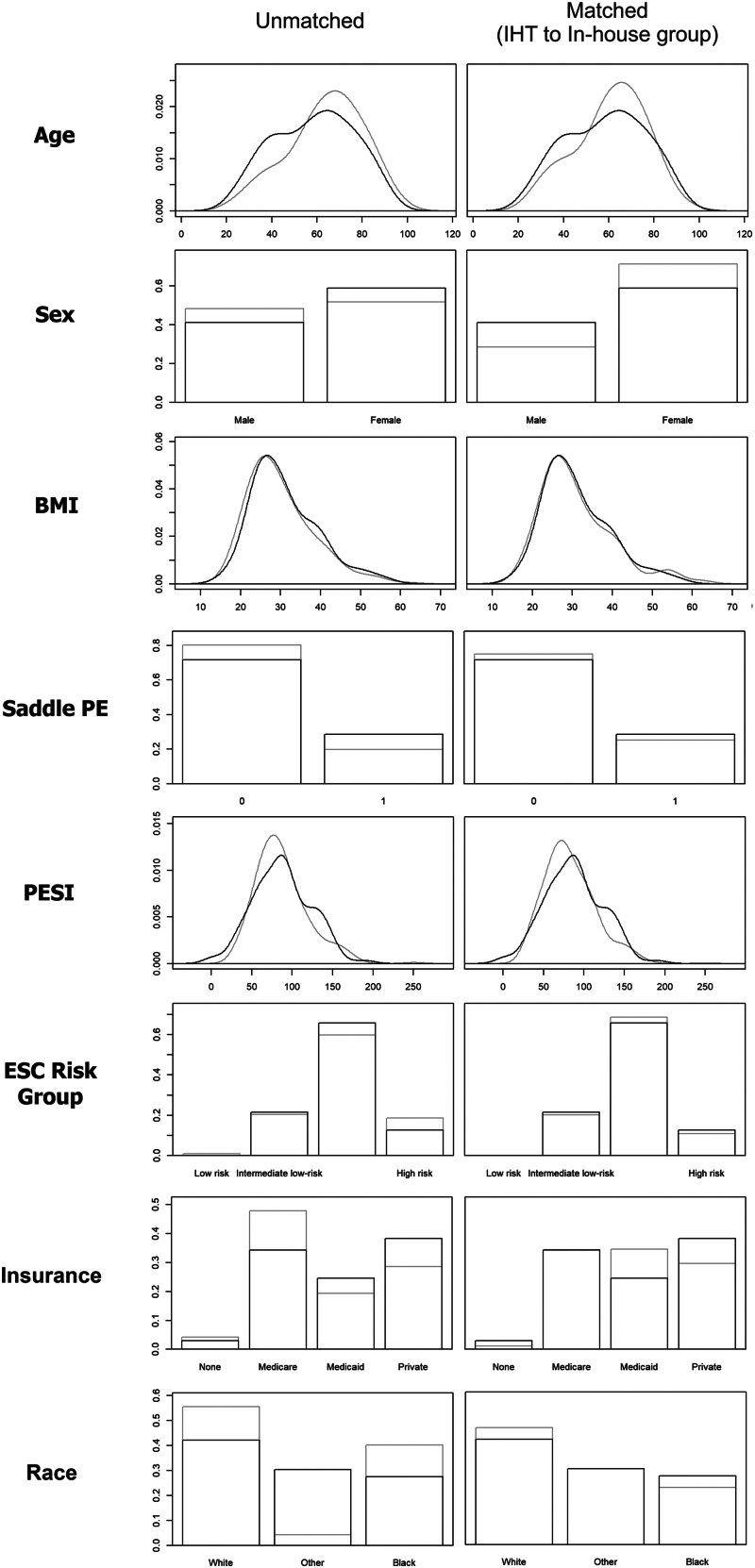
Density plot of the unmatched sample and matched sample (interhospital transfer [IHT] patients matched to patients treated in-house). Propensity score weighting was performed using the optimal full matching specification in the *MatchIt* package in R. Propensity scores were calculated using generalized linear regression with a *probit* link function from the following variables: age, sex, body mass index (BMI), Pulmonary Embolism Severity Index (PESI) score, European Society of Cardiology (ESC) risk group, presence of a saddle pulmonary embolus (PE), insurance, and race. The effective sample size was 154.52. Black color represents IHT patients, while gray color represents the control group.

To compare the outcomes of patients in the IHT and the in-house group, we performed a propensity score–weighted analysis using the in-house group as the control group. The IHT group of patients (*n* = 102; 26.4%) was matched to the control group (*n* = 284; 73.6%) on the propensity score using the optimal full matching specification. The density plot for this matched dataset is shown in Figure 2. Further balance measures are provided in Supplementary Table S1, and the Love plot is depicted in Supplementary Figure S1. While matching improved balance on most matching variables, imbalance was slightly increased for sex and Medicaid-insured patients. The effective sample size for this analysis was 154.5. Results of multivariable regression analyses in the propensity score–weighted dataset are provided in Table 3, while the results of multivariable regression analyses in the unmatched sample are provided in Supplementary Table S3. The odds of receiving systemic thrombolysis or advanced reperfusion therapies were not significantly different between the IHT and the in-house groups. However, patients in the IHT group had lower odds of 30-day mortality (odds ratio [OR], 0.88; *P* = .003) when compared with the in-house group. Moreover, patients in the IHT group had higher odds of 30-day major bleeding (OR, 1.03; *P* = .04) compared with the in-house group; however, odds of 30-day bleeding of any severity were not significantly different. The median hospital LOS for the IHT and the in-house groups were 5.9 (IQR, 3.2-10.9) and 6.2 (IQR, 3.7-10.5) days, respectively, which were not significantly different. The odds of 30-day readmission were not significantly different between the 2 groups as well. Interestingly, patients in the IHT group had lower odds of following up in primary care clinics (OR, 0.86; *P* = 0.04) than the in-house group, although they had higher odds of following up in pulmonary (OR, 1.16; *P* = .005) and hematology (OR, 1.19; *P* = .02) clinics than the control group.

**Table 3:** Comparison of receipt of advanced therapies and overall outcomes among the interhospital transfer group vs patients treated in-house.

Group	Systemic thrombolysis		
	Odds ratio	95% CI	*P* value
In-house	Reference	Reference	N/A
IHT	1.06	1.00-1.13	.056

Group	Catheter-directed thrombolysis		
	Odds ratio	95% CI	*P* value
In-house	Reference	Reference	N/A
IHT	0.96	0.92-1.01	.11

Group	Catheter-directed embolectomy		
	Odds ratio	95% CI	*P* value
In-house	Reference	Reference	N/A
IHT	1.00	0.91-1.09	.92

Group	Surgical embolectomy		
	Odds ratio	95% CI	*P* value
In-house	Reference	Reference	N/A
IHT	0.99	0.97- 1.01	.26

Group	Advanced reperfusion therapies		
	Odds ratio	95% CI	*P* value
In-house	Reference	Reference	N/A
IHT	0.95	0.86-1.06	.36

Group	In-hospital mortality		
	Odds ratio	95% CI	*P* value
In-house	Reference	Reference	N/A
IHT	0.95	0.89-1.02	.18

Group	Mortality at 30 d		
	Odds ratio	95% CI	*P* value
In-house	Reference	Reference	N/A
IHT	**0.88**	**0.81-0.96**	**.003**

Group	In-hospital bleeding of any severity		
	Odds ratio	95% CI	*P* value
In-house	Reference	Reference	N/A
IHT	1.02	0.94-1.10	.70

Group	In-hospital major bleeding		
	Odds ratio	95% CI	*P* value
In-house	Reference	Reference	N/A
IHT	**1.03**	**1.00-1.05**	**.04**

Group	Bleeding of any severity at 30 d		
	Odds ratio	95% CI	*P* value
In-house	Reference	Reference	N/A
IHT	1.02	0.95-1.09	.64

Group	Major bleeding at 30 d		
	Odds ratio	95% CI	*P* value
In-house	Reference	Reference	N/A
IHT	**1.03**	**1.00-1.05**	**.04**

Group	Length of stay (d)		
	IRR	95% CI	*P* value
In-house	Reference	Reference	N/A
IHT	0.07	0.0002-205	.51

Group	30 d readmission		
	Odds ratio	95% CI	*P* value
In-house	Reference	Reference	N/A
IHT	0.96	0.87-1.05	.34

Group	Primary care follow-up		
	Odds ratio	95% CI	*P* value
In-house	Reference	Reference	N/A
IHT	**0.86**	**0.75-0.99**	**.04**

Group	Pulmonary follow-up		
	Odds ratio	95% CI	*P* value
In-house	Reference	Reference	N/A
IHT	**1.16**	**1.05-1.29**	**.005**

Group	Hematology follow-up		
	Odds ratio	95% CI	*P* value
In-house	Reference	Reference	N/A
IHT	**1.19**	**1.03-1.37**	**.02**

*P* values and odds ratios were computed using multivariable quasi-binomial regression models within the propensity score–weighted sample.

Statistically significant results are shown in bold.

CDE, catheter-directed embolectomy; CDT, catheter-directed thrombolysis; IHT, interhospital transfer; IRR, incidence rate ratio; N/A, not applicable; SE, surgical embolectomy.

## 4 Discussion

The results of our study showed that in a cohort of patients with acute PE managed by PERT, patients treated in-house vs those who had undergone IHT had similar odds of receiving advanced reperfusion therapies, including CDT, CDE, and SE. Moreover, odds of receiving systemic thrombolysis did not differ significantly between the IHT and in-house groups either. However, patients in the IHT group had slightly lower odds of 30-day mortality and slightly higher odds of major bleeding, although hospital LOS and rates of 30-day readmission were not significantly different between the 2 groups. The results of our study stand in contrast with the results from analyses based on the Nationwide Inpatient Sample and National Readmissions Database [7,9], in which patients who underwent IHT were more likely to receive advanced therapies.

The odds of receiving advanced reperfusion therapies were not significantly different between the IHT and the in-house groups in our study. CDT, CDE, and SE were administered to 5.3%, 9.9%, and 0.3% of patients in the IHT group and 2.0%, 14.7%, and 6.9% of patients in the in-house group, respectively. These differences were not statistically significant in either univariable analyses in the unmatched sample (Supplementary Table S2) or regression analyses in the matched sample (Table 3). Moreover, rates of systemic thrombolysis for the IHT (8.8%) and in-house groups (4.6%) were also not significantly different. Contrary to our findings, Sedhom et al. [9] analyzed the Nationwide Readmissions Database between 2016 and 2019 and observed that patients undergoing IHT received advanced reperfusion therapies more often than other patients. Similar findings were reported by Carroll et al. [7], who retrospectively studied 2050 patients with acute PE and reported that advanced therapies were more frequently administered to patients undergoing IHT. In both studies, patients in the IHT group had higher risk of acute PE and were sicker than patients in the comparative groups [7,9]. Given that we performed a propensity score–weighted analysis that adjusted for patients’ severity of PE, we did not observe any significant association between receipt of advanced reperfusion therapies and IHT.

In our study, the odds of 30-day mortality were lower among patients who had undergone IHT than among those treated in-house. However, it should be noted that the rate of PE-related in-hospital mortality (2.9% in the IHT group vs 2.8% in the in-house group), as well as overall in-hospital mortality (4.9% in the IHT group vs 7% in the in-house group), was similar between the 2 groups. This may suggest that the lower 30-day mortality in the IHT group may be related to conditions other than acute PE, although there were no significant differences in the Charlson Comorbidity Index between the 2 groups. Moreover, the rates of in-hospital (6.5%) and 30-day mortality (9.6%) observed in our cohort were comparable with those reported in other published studies of acute PE patients managed by PERT [8,9,17]. In our study, patients who had undergone IHT had lower 30-day mortality when compared with patients treated in-house. Conversely, other studies in the literature have suggested that patients undergoing IHT tend to have similar rates of in-hospital and 30-day mortality [9,10,18]. Moreover, Elkaryoni et al. [8] studied the outcomes of acute PE patients within the Nationwide Inpatient Sample and found that patients requiring IHT had a higher adjusted rate of in-hospital mortality (OR, 1.27). However, within a subgroup analysis, they observed that among patients who received intervention, in-hospital mortality was not significantly different for transferred patients [8]. This explains why the results of our study were divergent from those of Elkaryoni et al. [8]; in our study, the odds of advanced reperfusion therapies were not significantly different for the IHT and in-house groups. Moreover, in a retrospective study of 2050 patients with acute PE, Carroll et al. [7] found that definite PE-related mortality was higher in the IHT group (38.5% vs 9.4%). This is in stark contrast to our study, where the in-hospital mortality in the IHT group was 4.9% compared with 7% for the in-house group. This discrepancy could be accounted for by the fact that the patients who underwent IHT in the study by Carroll et al. [7] were much sicker compared with the IHT group of patients in our study. Moreover, we used a propensity score–weighted analysis, which adjusted for the severity of PE and disease characteristics in the final analysis.

Patients who underwent IHT in our study had slightly higher odds of 30-day major bleeding compared with patients treated in-house. The rates of 30-day major bleeding in the IHT and in-house groups were 2.1% and 2.9%, respectively, which were both lower when compared with those reported in the published literature [7,9,10,18]. In a retrospective study of 267 patients, Barnett et al. [18] reported that the rates of 30-day major bleeding were 7.8% in the IHT group and 8.2% in the direct admit group. Similar results were reported by Beyer et al. [10], who observed rates of major bleeding of 5.7% and 8.6% in their control (direct admit) and IHT groups, respectively. Therefore, when viewed in context with the published literature, the observed overall rates of 30-day major bleeding in our study were much lower – even though the odds of major bleeding were higher in the IHT group.

We did not observe any significant associations between hospital LOS and the rate of 30-day readmission between the IHT and the in-house groups. The median hospital LOS for the IHT and in-house groups was 5.9 (IQR, 3.2-10.9) days and 6.2 (IQR, 3.7-10.5) days, respectively. The median hospital LOS for our study cohort was comparable with that reported by Barnett et al. [18], who observed a median hospital LOS of 3.8 (IQR, 2.4-7.9) days and 4.9 (IQR, 2.4-9.8) days in the IHT and direct admit groups, respectively. On the other hand, Sedhom et al. [9] investigated the National Readmissions Database and observed a hospital LOS of 13 (IQR, 6-23) days for IHT patients, which was significantly longer than that for the direct admit group (6 [IQR, 2-12] days). This discrepancy may be explained by the high acuity of patients included in their IHT group; for instance, 33.3% of patients in that group sustained cardiac arrest [9], which is much higher than that of our study. Moreover, we also did not observe any significant difference in the 30-day readmission rate between the IHT (9.8%) and the in-house (13%) groups. Overall, the 30-day readmission rate in our study (12.2%) was comparable with that reported by Murthi et al. [19] using the National Readmission Database—11.8% in 2018. In a retrospective single-center study, Beyer et al. [10] reported higher 90-day readmission rate of 26.8% in the IHT group compared with 15.8% in the direct admit group. Further research is needed to clarify the association of readmission rate with IHT among patients with acute PE.

The rates of follow-up after discharge differed significantly between the IHT and the in-house groups in our study. Patients in the IHT group had lower odds of following up in primary care clinics and higher odds of following up in specialty clinics after discharge compared with the in-house group. Timely outpatient care for patients with acute PE is crucial to assess the development of post-PE syndrome, and experts recommend a short-interval follow-up at 2 weeks to 3 months [20]. The overall rates of follow-up in primary care (71.2%), hematology (20.5%), and pulmonary clinics (30.3%) were comparable with real-world data reported by other investigators [17,21]. One of the determinants of early follow-up has been previously reported to be PE severity and a shorter hospital LOS. However, in the present study, we performed a propensity score–weighted analysis to control for PE severity. Moreover, the hospital LOS did not differ significantly between the IHT and the in-house groups.

The baseline clinical features and demographics of our patient cohort need to be considered when comparing the results of our study to the published literature. We performed a propensity score–weighted analysis that adjusted for age, sex, race, insurance status, PESI score, ESC risk group, saddle PE, and BMI. We did not observe any differences between the IHT and the in-house groups in univariable analyses with respect to demographics and baseline clinical characteristics. This was in contrast to the results reported by other similar studies [7,9,10], which observed that patients undergoing IHT had higher PESI scores, ESC risk group, and higher frequency of saddle PE and RV strain. Other published studies have reported that Black patients and those with Medicaid insurance or self-paying status were less likely to be transferred compared with other patients [22,23]. Moreover, studies analyzing the association of race and insurance status with outcomes of acute PE patients managed by PERT have reported that Black, uninsured, and economically disadvantaged patients undergo fewer advanced therapies and have higher odds of mortality, bleeding, and readmission compared with other patients [24,25].

Our study is not without limitations. We recognize that a smaller sample size may have contributed to a reduction in power, especially when reporting differences in intervention rates between the 2 groups, while other larger cohorts have shown that IHT patients tended to receive interventions more often [7,9,10]. Along with a smaller sample size, our patients were perhaps not as critically ill as those in some of the other studies [8,9]. Moreover, we included many patients who had undergone IHT from affiliated hospitals within the Mount Sinai Health System; this could have limited the generalizability of our results. Additionally, we did not compare process metrics (door-to-PERT and door-to-intervention times) between the 2 groups, which could have been associated with the receipt of therapies or overall outcomes of patients. Lastly, some imbalance remained in the matching variables, including sex and Medicaid insurance, which suggests that some residual confounding persisted, even among the variables used for matching. Perhaps this was a result of the small sample size and some degree of underlying nonexchangeability of the 2 comparator groups. Moreover, given the observational and nonrandomized nature of this study, the possibility of unmeasured confounding remains as well. Future research should focus on exploring the associations between process metrics and overall outcomes of acute PE patients undergoing IHT.

## 5 Conclusion

Within a cohort of acute PE patients managed by PERT, patients who had been transferred from other hospitals had lower odds of 30-day mortality and higher odds of 30-day major bleeding when compared with acute PE patients managed in-house. The odds of receiving systemic thrombolysis or advanced reperfusion therapies were not significantly different between the IHT and the in-house cohorts. Hospital LOS and 30-day readmission rates were not significantly different between the 2 groups of patients. These results suggest that PERT-guided IHT for patients with acute PE may provide outcomes comparable with those of patients presenting directly to hospitals with PERT capabilities.

## Figures and Tables

**Supplementary Figure 1:**