# Supplementary material for: Outcomes of patients with acute pulmonary embolism managed in-house vs those transferred between hospitals: a retrospective observational study
Source: Res Pract Thromb Haemost. 2024 Oct 29;8(8):102606. doi: 10.1016/j.rpth.2024.102606 (PMC11647229; doi:10.1016/j.rpth.2024.102606)
Supplement: Online Supplement Data [file mmc1.docx]

*Original Research*

**Outcomes of patients with acute pulmonary embolism managed in-house versus those transferred between hospitals: A retrospective observational study**

Priyanka Sridhar^1^ (MD), Hong Yu Wang^1^ (MD), Agostina Velo^1^ (MD), Destiny Nguyen^1^ (MD), Avinash Singh^2^ (MBBS), Abdul Rehman^3^ (MBBS), Jason Filopei^2^ (MD), Madeline Ehrlich^2^ (MPharm), Robert Lookstein^4^ (MD), David J. Steiger^2*^ (MD)

*^1^ Department of Medicine, Icahn School of Medicine at Mount Sinai Health System, New York City, New York 10029, United States ^2^ Division of Pulmonary, Critical Care and Sleep Medicine, Department of Medicine, Icahn School of Medicine at Mount Sinai Health System, New York City, New York 10029, United States*

*^3^ Department of Medicine, TidalHealth Peninsula Regional, Salisbury, Maryland 21801, United States*
*^4^ Department of Medicine, Icahn School of Medicine at Mount Sinai Health System, New York City, New York 10029, United States*

* Corresponding author

David J. Steiger, MD

Professor and Division Chief,

Division of Pulmonary, Critical Care, and Sleep Medicine,

Department of Medicine,

Mount Sinai Beth Israel,

Icahn School of Medicine at Mount Sinai,

New York City, NY 10019,
United States of America.

Tel: (212) 420-2377

david.steiger@mountsinai.org

**
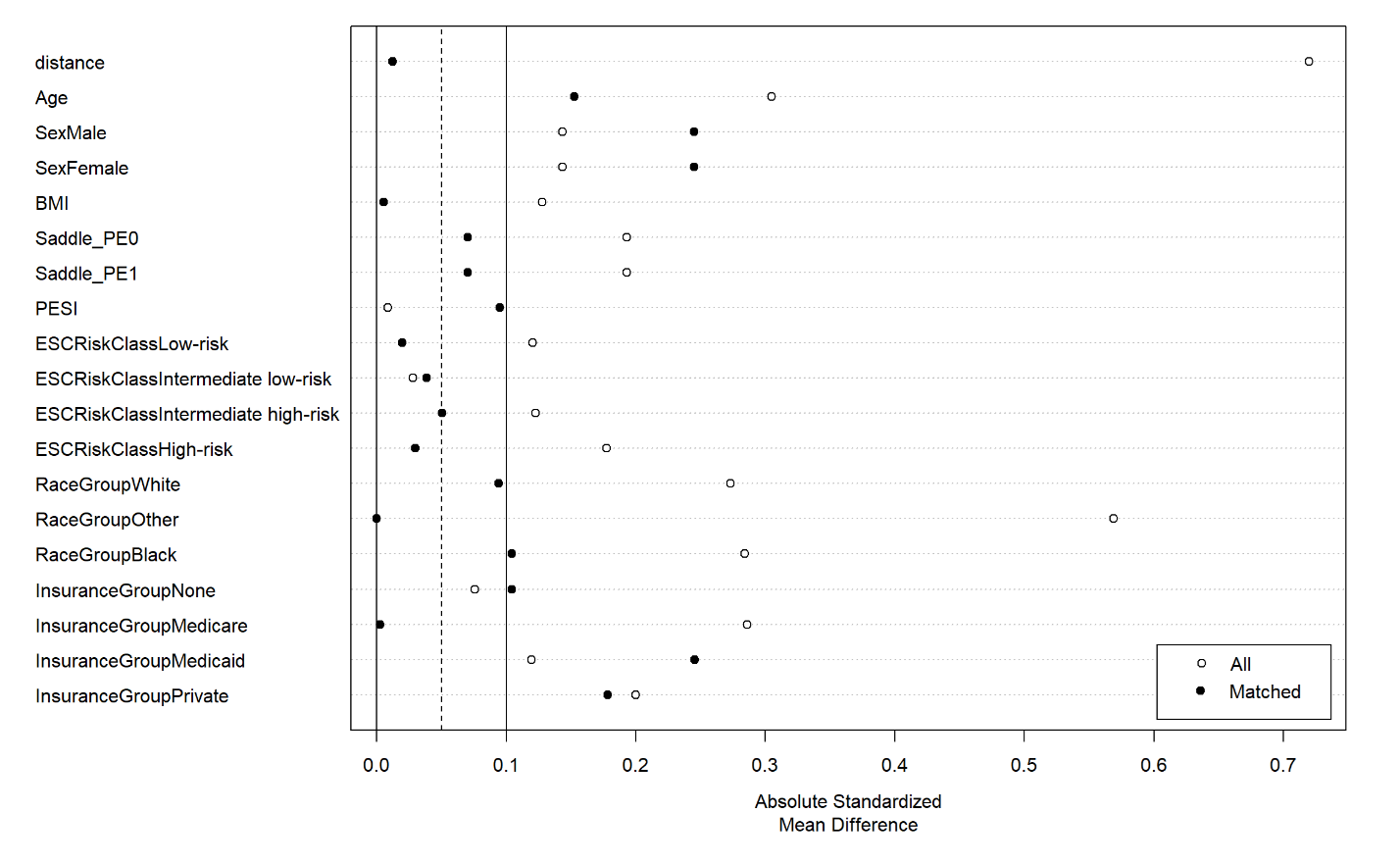
**

**Supplementary Figure S1:** Love plot depicting the standardized mean differences of matching variables between the unmatched and the matched sample

**Supplementary Table S1:** Balance measures before and after optimal full matching in the study sample

| **UNMATCHED SAMPLE** | | | | | | |
| --- | --- | --- | --- | --- | --- | --- |
| **Variable** | **Means (treated)** | **Means (control)** | **SMD** | **Variance ratio** | **eCDF mean** | **eCDF maximum** |
| Propensity score | 0.3974 | 0.2178 | 0.7198 | 3.7663 | 0.2285 | 0.3754 |
| Age | 58.5 | 63.8979 | -0.3049 | 1.1303 | 0.0727 | 0.1628 |
| Sex: Male | 0.4118 | 0.4824 | -0.1435 | N/A | 0.0706 | 0.0706 |
| Sex: Female | 0.5882 | 0.5176 | 0.1435 | N/A | 0.0706 | 0.0706 |
| BMI | 31.0410 | 29.9780 | 0.1277 | 1.0163 | 0.0403 | 0.0941 |
| Saddle PE: no | 0.7157 | 0.8028 | -0.1932 | N/A | 0.0871 | 0.0871 |
| Saddle PE: yes | 0.2843 | 0.1972 | 0.1932 | N/A | 0.0871 | 0.0871 |
| PESI | 87.1961 | 86.8908 | 0.0086 | 1.2002 | 0.0421 | 0.1144 |
| ESC-low | 0.0000 | 0.0106 | -0.1205 | N/A | 0.0106 | 0.0106 |
| ESC-ILR | 0.2157 | 0.2042 | 0.0279 | N/A | 0.0115 | 0.0115 |
| ESC-IHR | 0.6569 | 0.5986 | 0.1227 | N/A | 0.0583 | 0.0583 |
| ESC-high | 0.1275 | 0.1866 | -0.1774 | N/A | 0.0592 | 0.0592 |
| Race: White | 0.4216 | 0.5563 | -0.2729 | N/A | 0.1348 | 0.1348 |
| Race: Other | 0.3039 | 0.0423 | 0.5689 | N/A | 0.2617 | 0.2617 |
| Race: Black | 0.2745 | 0.4014 | -0.2844 | N/A | 0.1269 | 0.1269 |
| Insurance: None | 0.0294 | 0.0423 | -0.0760 | N/A | 0.0128 | 0.0128 |
| Insurance: Medicare | 0.3431 | 0.4789 | -0.2589 | N/A | 0.1357 | 0.1357 |
| Insurance: Medicaid | 0.2451 | 0.1937 | 0.1196 | N/A | 0.0514 | 0.0514 |
| Insurance: Private | 0.3824 | 0.2852 | 0.1999 | N/A | 0.0971 | 0.0971 |
| **MATCHED SAMPLE** | | | | | | |
| **Variable** | **Means (treated)** | **Means (control)** | **SMD** | **Variance ratio** | **eCDF mean** | **eCDF maximum** |
| Propensity score | 0.3974 | 0.3943 | 0.0123 | 1.0281 | 0.0056 | 0.0588 |
| Age | 58.5 | 61.2042 | -0.1528 | 1.2807 | 0.0458 | 0.1455 |
| Sex: Male | 0.4118 | 0.2911 | 0.2452 | N/A | 0.1207 | 0.1207 |
| Sex: Female | 0.5882 | 0.7089 | -0.2452 | N/A | 0.1207 | 0.1207 |
| BMI | 31.0410 | 30.9946 | 0.0056 | 0.8440 | 0.0195 | 0.0681 |
| Saddle PE: no | 0.7157 | 0.7473 | -0.0702 | N/A | 0.0317 | 0.0317 |
| Saddle PE: yes | 0.2843 | 0.2527 | 0.0702 | N/A | 0.0317 | 0.0317 |
| PESI | 87.1961 | 83.8386 | 0.0950 | 1.2606 | 0.0450 | 0.1434 |
| ESC-low | 0.0000 | 0.0017 | -0.0197 | N/A | 0.0017 | 0.0017 |
| ESC-ILR | 0.2157 | 0.1998 | 0.0385 | N/A | 0.0158 | 0.0158 |
| ESC-IHR | 0.6569 | 0.6809 | -0.0506 | N/A | 0.0240 | 0.0240 |
| ESC-high | 0.1275 | 0.1176 | 0.0296 | N/A | 0.0099 | 0.0099 |
| Race: White | 0.4216 | 0.4681 | -0.0942 | N/A | 0.0465 | 0.0465 |
| Race: Other | 0.3039 | 0.3039 | 0.0000 | N/A | 0.0000 | 0.0000 |
| Race: Black | 0.2745 | 0.2280 | 0.1042 | N/A | 0.0465 | 0.0465 |
| Insurance: None | 0.0294 | 0.0118 | 0.1045 | N/A | 0.0177 | 0.0177 |
| Insurance: Medicare | 0.3431 | 0.3419 | 0.0027 | N/A | 0.0013 | 0.0013 |
| Insurance: Medicaid | 0.2451 | 0.3506 | -0.2454 | N/A | 0.1055 | 0.1055 |
| Insurance: Private | 0.3824 | 0.2957 | 0.1783 | N/A | 0.0866 | 0.0866 |

*BMI*=Body mass index; *eCDF*=empirical cumulative distribution function; *ESC*=European Society of Cardiology; *IHR*=intermediate high-risk; *ILR*=intermediate low-risk; *PESI*=Pulmonary Embolism Severity Index; *N/A*=not applicable; *SMD*=standardized mean difference.

**Supplementary Table S2:** Univariable analyses for comparison of baseline characteristics between the IHT and in-house groups

| **Variable** | **Test statistic** | ***p*-value*** |
| --- | --- | --- |
| Age | W=16931 | 0.1443 |
| Sex | *χ^2^*=1.2348, *df*=1 | 0.2665 |
| **Race** | Fisher’s exact test | **<0.0001** |
| Insurance | Fisher’s exact test | 0.1137 |
| Body mass index | W=13300 | 0.2206 |
| Prior episode of VTE | *χ^2^*=0.0010256, *df*=1 | 0.9745 |
| Prior anticoagulation | *χ^2^*=1.7501, *df*=1 | 0.1859 |
| Charlson Comorbidity Index | W=15410 | 0.335 |
| History of cardiac disease | *χ^2^*=1.3505, *df*=1 | 0.2452 |
| History of asthma or COPD | *χ^2^*=0.012786, *df*=1 | 0.91 |
| History of other lung disease | Fisher’s exact test | 0.105 |
| Pulmonary hypertension | Fisher’s exact test | 0.09 |
| Active malignancy | *χ^2^*=4.8633, *df*=1 | 0.07 |
| Immobility for ≥ 3 days | *χ^2^*=0.0001, *df*=1 | 0.999 |
| Surgery within 4 weeks | *χ^2^*=3.8310, *df*=1 | 0.06 |
| Active smoker | *χ^2^*=1.782, *df*=1 | 0.1819 |
| Predisposing medications | *χ^2^*=5.4453, *df*=1 | 0.201 |
| Recent long flight or travel | *χ^2^*=1.9047, *df*=1 | 0.1676 |
| Pleuritic chest pain | *χ^2^*=3.7710, *df*=1 | 0.077 |
| Hemoptysis | Fisher’s exact test | 0.5544 |
| Dyspnea | *χ^2^*=9.3006, *df*=1 | 0.0923 |
| Syncope | *χ^2^*=1.0964, *df*=1 | 0.2951 |
| Unilateral leg swelling | *χ^2^*=0.0001, *df*=1 | 0.999 |
| Tachycardia | *χ^2^*=3.7820, *df*=1 | 0.06 |
| Hypotension | Fisher’s exact test | 0.07 |
| Tachypnea | *χ^2^*=1.3735, *df*=1 | 0.2412 |
| **Hypoxia** | *χ^2^*=47.94, *df*=1 | **<0.0001** |
| PESI score | W=13927 | 0.5647 |
| ESC risk group | Fisher’s exact test | 0.4651 |
| Site where PE diagnosed | *χ^2^*=0.25304, *df*=1 | 0.6149 |
| **Disposition on admission** | Fisher’s exact test | **<0.0001** |
| Central PE | *χ^2^*=1.3808, *df*=1 | 0.24 |
| Saddle PE | *χ^2^*=2.8299, *df*=1 | 0.09253 |
| RV dilatation on CT | *χ^2^*=2.8299, *df*=1 | 0.5604 |
| Echocardiography performed | Fisher’s exact test | 0.5377 |
| RV dysfunction on TTE | *χ^2^*=0.16992, *df*=1 | 0.6802 |
| Concomitant DVT | *χ^2^*=1.7113, *df*=1 | 0.1908 |
| D-dimer | W=6766.5 | 0.1823 |
| Troponin I | W=18209 | 0.7718 |
| Brain natriuretic peptide | W=12592 | 0.1028 |

* *p*-values adjusted for multiple comparisons using the Hochberg method (1988) and computed from either: (a) Chi-square (*χ^2^*) test of independence with Yates continuity correction in the case of qualitative variables; (b) Fisher’s exact test with continuity correction for qualitative variables if the expected frequency in any cell was less than 5; or (c) Wilcoxon rank-sum test with continuity correction in the case of quantitative variables. All statistical tests were two-tailed.

*COPD*=Chronic obstructive pulmonary disease; *CT=*computed tomography; *df*=degrees of freedom; *DVT*=deep venous thrombosis; *ESC*=European Society of Cardiology; *IHT*=interhospital transfer; *PE*=pulmonary embolism; *PESI*=PE Severity Index; *RV*=right ventricle; *TTE*=transthoracic echocardiography; *VTE*=venous thromboembolism.

**Supplementary Table S3:** Multivariable regression models in the unmatched sample comparing receipt of advanced therapies and overall outcomes among the IHT group versus patients treated in-house

| **GROUP** | **SYSTEMIC THROMBOLYSIS** | | | |
| --- | --- | --- | --- | --- |
|  | **Events** | **Odds ratio*** | **95% CI** | ***p*-value*** |
| In-house (n=284) | 13 | Reference | Reference | N/A |
| IHT (n=102) | 9 | 2.124 | 0.691 to 6.173 | 0.173 |
| **GROUP** | **CATHETER-DIRECTED THROMBOLYSIS** | | | |
|  | **Events** | **Odds ratio*** | **95% CI** | ***p*-value*** |
| In-house (n=284) | 15 | Reference | Reference | N/A |
| IHT (n=102) | 2 | 0.369 | 0.019 to 1.000 | 0.051 |
| **GROUP** | **CATHETER-DIRECTED EMBOLECTOMY** | | | |
|  | **Events** | **Odds ratio*** | **95% CI** | ***p*-value*** |
| In-house (n=284) | 28 | Reference | Reference | N/A |
| IHT (n=102) | 15 | 1.208 | 0.541 to 2.58 | 0.633 |
| **GROUP** | **SURGICAL EMBOLECTOMY** | | | |
|  | **Events** | **Odds ratio*** | **95% CI** | ***p*-value*** |
| In-house (n=284) | 7 | Reference | Reference | N/A |
| IHT (n=102) | 1 | 0.241 | 0.039 to 1.031 | 0.093 |
| **GROUP** | **ADVANCED REPERFUSION THERAPIES** | | | |
|  | **Events** | **Odds ratio*** | **95% CI** | ***p*-value*** |
| In-house (n=284) | 49 | Reference | Reference | N/A |
| IHT (n=102) | 18 | 0.707 | 0.340 to 1.401 | 0.335 |
| **GROUP** | **IN-HOSPITAL MORTALITY** | | | |
|  | **Events** | **Odds ratio*** | **95% CI** | ***p*-value*** |
| In-house (n=284) | 20 | Reference | Reference | N/A |
| IHT (n=102) | 5 | 0.463 | 0.120 to 1.453 | 0.221 |
| **GROUP** | **MORTALITY AT THIRTY DAYS** | | | |
|  | **Events** | **Odds ratio*** | **95% CI** | ***p*-value*** |
| In-house (n=284) | 30 | Reference | Reference | N/A |
| IHT (n=102) | 7 | **0.383** | **0.120 to 0.868** | **0.009** |
| **GROUP** | **IN-HOSPITAL BLEEDING OF ANY SEVERITY** | | | |
|  | **Events** | **Odds ratio*** | **95% CI** | ***p*-value*** |
| In-house (n=284) | 22 | Reference | Reference | N/A |
| IHT (n=102) | 10 | 1.560 | 0.605 to 3.805 | 0.339 |
| **GROUP** | **IN-HOSPITAL MAJOR BLEEDING** | | | |
|  | **Events** | **Odds ratio*** | **95% CI** | ***p*-value*** |
| In-house (n=284) | 5 | Reference | Reference | N/A |
| IHT (n=102) | 3 | 0.241 | 0.039 to 0.968 | 0.073 |
| **GROUP** | **BLEEDING OF ANY SEVERITY AT THIRTY DAYS** | | | |
|  | **Events** | **Odds ratio*** | **95% CI** | ***p*-value*** |
| In-house (n=284) | 25 | Reference | Reference | N/A |
| IHT (n=102) | 11 | 1.311 | 0.259 to 5.477 | 0.723 |
| **GROUP** | **MAJOR BLEEDING AT THIRTY DAYS** | | | |
|  | **Events** | **Odds ratio*** | **95% CI** | ***p*-value*** |
| In-house (n=284) | 6 | Reference | Reference | N/A |
| IHT (n=102) | 3 | 1.233 | 0.488 to 2.936 | 0.644 |
| **GROUP** | **LENGTH OF STAY (days)** | | | |
|  | **Median (IQR)** | **IRR†** | **95% CI** | ***p*-value†** |
| In-house (n=284) | 6.2 (3.7‒10.5) | Reference | Reference | N/A |
| IHT (n=102) | 5.9 (3.2‒10.9) | 0.067 | 0.0002 to 205 | 0.509 |
| **GROUP** | **THIRTY-DAY RE-ADMISSION** | | | |
|  | **Events** | **Odds ratio*** | **95% CI** | ***p*-value*** |
| In-house (n=284) | 37 | Reference | Reference | N/A |
| IHT (n=102) | 10 | 0.625 | 0.254 to 1.399 | 0.277 |
| **GROUP** | **PRIMARY CARE FOLLOW-UP** | | | |
|  | **Events** | **Odds ratio*** | **95% CI** | ***p*-value*** |
| In-house (n=284) | 216 | Reference | Reference | N/A |
| IHT (n=102) | 59 | **0.293** | **0.163 to 0.522** | **<0.001** |
| **GROUP** | **PULMONARY FOLLOW-UP** | | | |
|  | **Events** | **Odds ratio*** | **95% CI** | ***p*-value*** |
| In-house (n=284) | 75 | Reference | Reference | N/A |
| IHT (n=102) | 42 | **1.670** | **1.014 to 2.129** | **0.043** |
| **GROUP** | **HEMATOLOGY FOLLOW-UP** | | | |
|  | **Events** | **Odds ratio*** | **95% CI** | ***p*-value*** |
| In-house (n=284) | 41 | Reference | Reference | N/A |
| IHT (n=102) | 38 | **3.000** | **1.626 to 5.542** | **<0.001** |

* *p*-values and odds ratios computed using multivariable logistic regression models constructed within the unmatched sample incorporating matching variables as covariates (in addition to IHT *vs.* control group membership)

† *p*-value and incidence rate ratio computed using negative binomial regression model built within the unmatched sample incorporating matching variables as covariates (in addition to IHT *vs.* control group membership)

*CI*=confidence interval; *IHT*=interhospital transfer; *IQR*=interquartile range; *IRR*=incidence rate ratio; *N/A*=not applicable.
